# Epidemiology and Prognosis of Coagulase-Negative Staphylococcal Endocarditis: Impact of Vancomycin Minimum Inhibitory Concentration

**DOI:** 10.1371/journal.pone.0125818

**Published:** 2015-05-11

**Authors:** Cristina García de la Mària, Carlos Cervera, Juan M. Pericàs, Ximena Castañeda, Yolanda Armero, Dolors Soy, Manel Almela, Salvador Ninot, Carlos Falces, Carlos A. Mestres, Jose M. Gatell, Asuncion Moreno, Francesc Marco, José M. Miró, Hospital Clinic Endocarditis Study Group

**Affiliations:** 1 Infectious Diseases Service, The Hospital Clínic, Institut d’Investigacions Biomèdiques August Pi i Sunyer (IDIBAPS), University of Barcelona School of Medicine, Barcelona, Spain; 2 Microbiology Service, The Hospital Clínic, Institut d’Investigacions Biomèdiques August Pi i Sunyer (IDIBAPS), University of Barcelona School of Medicine, Barcelona, Spain; 3 Pharmacy Service, The Hospital Clínic, Institut d’Investigacions Biomèdiques August Pi i Sunyer (IDIBAPS), University of Barcelona School of Medicine, Barcelona, Spain; 4 Department of Cardiovascular Surgery, The Hospital Clínic, Institut d’Investigacions Biomèdiques August Pi i Sunyer (IDIBAPS), University of Barcelona School of Medicine, Barcelona, Spain; 5 Cardiology Service, The Hospital Clínic, Institut d’Investigacions Biomèdiques August Pi i Sunyer (IDIBAPS), University of Barcelona School of Medicine, Barcelona, Spain; Leibniz-Institute DSMZ, GERMANY

## Abstract

This study describes coagulase-negative staphylococcal (CoNS) infective endocarditis (IE) epidemiology at our institution, the antibiotic susceptibility profile, and the influence of vancomycin minimum inhibitory concentration (MIC) on patient outcomes. One hundred and three adults with definite IE admitted to an 850-bed tertiary care hospital in Barcelona from 1995-2008 were prospectively included in the cohort. We observed that CoNS IE was an important cause of community-acquired and healthcare-associated IE; one-third of patients involved native valves. *Staphylococcus epidermidis* was the most frequent species, methicillin-resistant in 52% of patients. CoNS frozen isolates were available in 88 patients. Vancomycin MICs of 2.0 μg/mL were common; almost all cases were found among *S*. *epidermidis* isolates and did not increase over time. Eighty-five patients were treated either with cloxacillin or vancomycin: 38 patients (Group 1) were treated with cloxacillin, and 47 received vancomycin; of these 47, 27 had CoNS isolates with a vancomycin MIC <2.0 μg/mL (Group 2), 20 had isolates with a vancomycin MIC ≥2.0 μg/mL (Group 3). One-year mortality was 21%, 48%, and 65% in Groups 1, 2, and 3, respectively (*P*=0.003). After adjusting for confounders and taking Group 2 as a reference, methicillin-susceptibility was associated with lower 1-year mortality (OR 0.12, 95% CI 0.02-0.55), and vancomycin MIC ≥2.0 μg/mL showed a trend to higher 1-year mortality (OR 3.7, 95% CI 0.9-15.2; *P*=0.069). Other independent variables associated with 1-year mortality were heart failure (OR 6.2, 95% CI 1.5-25.2) and pacemaker lead IE (OR 0.1, 95%CI 0.02-0.51). In conclusion, methicillin-resistant *S*.*epidermidis* was the leading cause of CoNS IE, and patients receiving vancomycin had higher mortality rates than those receiving cloxacillin; mortality was higher among patients having isolates with vancomycin MICs ≥2.0 μg/mL.

## Introduction

Coagulase-negative staphylococci (CoNS) have come to be recognized as important, commonly isolated pathogens [1,2]. Infections are usually associated with healthcare settings and occur in patients harbouring indwelling polymer or metallic devices [3]. CoNS cause >10% of all infective endocarditis (IE) cases [4] and are among the most frequent etiological agents of intracardiac prosthetic device infections, such as prosthetic valve endocarditis (PVE) and intracardiac device (ICD) endocarditis [5–7]. In addition, these microorganisms are becoming an important cause of native valve endocarditis (NVE) [8]. Among CoNS species, *Staphylococcus lugdunensis* is notable for its particular virulence [9].

Resistance to methicillin and other antibiotics is becoming more frequent among CoNS. A glycopeptide, such as vancomycin, is the recommended treatment for methicillin-resistant CoNS (MR-CoNS) NVE, while gentamicin and rifampin are typically added in PVE [10]. The emergence of CoNS with reduced susceptibility to vancomycin [3], together with the increasing prevalence of glycopeptide-intermediate *Staphylococcus epidermidis* (GISE) [11] and resistance to rifampin and gentamicin among methicillin-resistant *S*. *epidermidis* (MRSE), limits therapeutic options and warrants investigation of alternative bactericidal agents.

Among patients with *Staphylococcus aureus* bacteremia, increased vancomycin minimum inhibitory concentrations (MICs) have been associated with clinical failures [12], while vancomycin MICs >1 μg/mL have been associated with higher mortality [13]. There currently are no data regarding the influence of vancomycin MIC on the outcome of CoNS IE.

This study aimed to characterize the epidemiology, clinical characteristics, and antibiotic susceptibility pattern of CoNS IE, and the influence of methicillin susceptibility and vancomycin MIC on outcomes.

## Methods

This prospective cohort study was performed in an urban tertiary care hospital with 850 beds in Barcelona, Spain. All consecutive CoNS IE patients seen from 1995 to 2008 were recorded in a database using a standardized case report form. Only patients with a definite diagnosis of IE [14] were included. All survivors were followed ≥1 year. The Ethics Committee of our institution gave its approval to perform the current study.

The variables analyzed, including age, gender, history of chronic disease, calendar year, right- vs. left-sided IE, type of endocarditis (NVE, PVE, or ICD-associated), place of acquisition (community-acquired, nosocomial, or non-nosocomial healthcare-associated) [15], clinical complications (heart failure, renal failure, or systemic emboli, including stroke), need for surgery, and in-hospital and 1-year mortality, have been previously defined [16].

Due to the duration of the study period, antimicrobial treatment for CoNS IE was given according to the 1995 (originally) and 2005 (later) American Heart Association (AHA) recommendations [10,17], both of which recommend the same agents for CoNS IE. Methicillin-susceptible CoNS (MS-CoNS) IE was treated with cloxacillin, and MR-CoNS IE was treated with either vancomycin alone (NVE) or combined with other antibiotics (PVE or ICD IE). The decision of using monotherapy or combination was at the discretion of the treating physician and influenced by factors related to patient’ clinical status (ie, renal function, drug allergy, potential drug interactions, comorbidity, age). Per guidelines of the time [10,17], a vancomycin trough concentration of 10–15 μg/mL was targeted.

In order to analyze the impact of methicillin resistance and vancomycin MIC on outcomes, we divided patients into 3 groups: patients treated with cloxacillin for MS-CoNS IE (Group 1), vancomycin for CoNS with vancomycin MIC <2 μg/mL (Group 2), or vancomycin for CoNS with vancomycin MIC ≥2 μg/mL (Group 3). In the event of polyclonal or polymicrobial CoNS IE, we categorized patients per the higher vancomycin MIC.

### Identification of CoNS Isolates

Isolates were stored at -80°C in skim milk. Isolates were identified using the API ID 32 Staph (bioMérieux, Marcy l'Etoile, France). Species were divided into 4 groups: *S*. *epidermidis*, *S*. *lugdunensis*, other CoNS, and polymicrobial IE. Polymicrobial infections were caused by different CoNS isolates. Polyclonal CoNS infections were caused by ≥2 isolates of the same species with different antibiotic susceptibilities and each isolate was counted separately.

### Antibiotic Susceptibility Testing

Susceptibility was determined by Etest according to the manufacturer’s recommendations (AB Biodisk-bioMérieux, Marcy l'Etoile, France). Etest strips were purchased from bioMérieux (Madrid, Spain). The following antimicrobials were evaluated: penicillin, oxacillin, erythromycin, clindamycin, gentamicin, ciprofloxacin, cotrimoxazole, rifampin, vancomycin, teicoplanin, linezolid, and daptomycin. The latter 2 agents were tested retrospectively after they became available and with emerging data about the impact of elevated vancomycin MICs. *S*. *aureus* ATCC 29213 was used as the test control strain. For vancomycin, isolates were divided according to MIC (<2 μg/mL or ≥2 μg/mL).

### Statistical Analyses

Categorical variables were summarized as percentages and compared using the Chi-square or Fisher’s exact test. Continuous variables were summarized as mean and SD. The Mantel-Haenszel test for trend was used if there were significant differences in vancomycin MIC over time among the isolates. The Kaplan-Meier estimator was used for survival analysis, and curves were compared using the log-rank test. For logistic regression analysis, predictors with a *P*<0.30 were included, and it was performed by a likelihood ratio-based backward exclusion method. A 2-sided *P*<0.05 was considered to be statistically significant. All statistics were calculated with SPSS statistical package version 16.0 (SPSS, Inc., Chicago, IL, USA).

## Results

### Clinical Characteristics of CoNS IE

**Fig 1:**
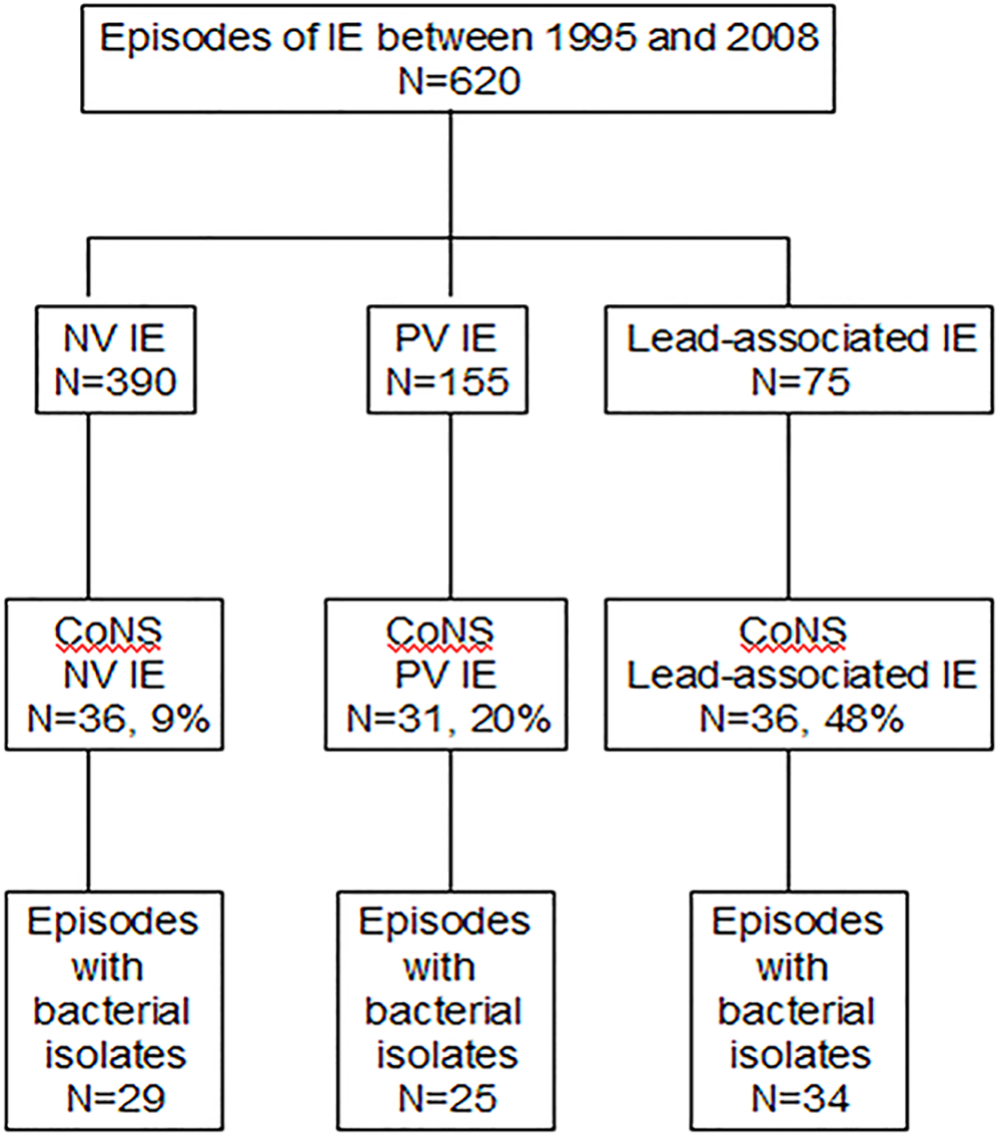
Patient disposition. Abbreviations: IE, infective endocarditis; NV, native valve; PV, prosthetic valve; CoNS, coagulase-negative staphylococci.

There were 103 patients with CoNS IE during the study, representing 16% of the 620 IE cases diagnosed at our institution (Fig 1). Regarding the type of IE, 36 (35%) were ICD-associated, 31 (30%) were PVE, and 36 (35%) were NVE. Stored CoNS isolates were available for 88 patients, in whom 98 isolates were identified. Isolates could not be obtained in the others due to transfer from another institution.

The clinical characteristics and outcomes of these 103 patients are summarized by type of endocarditis in Table 1. The majority of infections were due to *S*. *epidermidis*, while the species was not identified in 15 (15%) patients. In 59 (57%) patients, isolates were susceptible to methicillin, with a lower rate in PVE (36%) compared with NVE (61%) or ICD IE (72%) (*P* = 0.009). The aortic valve was most commonly involved, but 13 (13%) patients had involvement of multiple valves. While vegetations were present in 82 (80%) patients, only 13 (13%) patients had evidence of systemic emboli. Median vegetation size was greatest in ICD IE (*P* = 0.007); perivalvular abscesses were most common among patients with PVE (*P*<0.001). Sixty-six patients (64%) required surgery, including almost all patients with ICD IE. Mortality rates were similar within the NVE and PVE groups and lowest among patients with ICD IE (*P*<0.001).

**Table 1:** Clinical characteristics and outcome of 103 consecutive patients with IE due to CoNS, according to the type of endocarditis (1995–2008).

		NVE (N = 36)	PVE (N = 31)	ICD IE (N = 36)	*P* value
Mean age (SD), y		61.8 (16.7)	60.7 (11.3)	62.1 (19.4)	0.506
Male gender		27 (75)	24 (77)	30 (83)	0.676
Year of diagnosis					0.560
	1995–1999	13 (36)	9 (29)	9 (25)	
	2000–2004	14 (39)	9 (29)	15 (42)	
	2005–2008	9 (25)	13 (42)	12 (33)	
CoNS species^a^					0.400
	*S*. *epidermidis*	17 (47)	18 (58)	25 (69)	
	*S*. *lugdunensis*	4 (11)	2 (7)	5 (14)	
	Other CoNS^b^	4 (11)	4 (13)	3 (8)	
	Polymicrobial	4 (11)	1 (3)	1 (3)	
	Unknown	7 (20)	6 (19)	2 (6)	
Methicillin susceptibility		22 (61)	11 (36)	26 (72)	0.009
Predisposing conditions and underlying diseases					
	Diabetes mellitus	1 (3)	8 (26)	4 (11)	0.015
	Chronic renal failure	4 (11)	2 (7)	1 (3)	0.386
	Hemodialysis	4 (11)	0	0	0.034
	History of cancer	4 (11)	5 (16)	2 (6)	0.239
	HIV infection	1 (3)	0	0	1.000
	Chronic liver disease	9 (25)	3 (10)	2 (6)	0.036
	Chronic lung disease	0	2 (7)	3 (8)	0.267
	Transplantation	1 (3)	0	0	1.000
Presumed mode of acquisition					0.004
	Nosocomial	11 (31)	4 (13)	6 (17)	
	Non-nosocomial health care associated	11 (31)	18 (58)	7 (19)	
	Community acquired	14 (39)	9 (29)	23 (64)	
Valve involvement					<0.001
	Aortic	14 (39)	12 (39)	1 (3)^c^	
	Mitral	13 (36)	11 (36)	0	
	Tricuspid	1 (3)	1 (3)	3 (8)^c^	
	PCM/ICD wire^c^	0	0	34 (94)	
	Unknown	1 (3)	1 (3)	0	
	Mitral + aortic	4 (11)	5 (16)	0	
	Tricuspid + aortic	2 (6)	0	0	
	Tricuspid + aortic + mitral	1 (3)	1 (3)	0	
Echocardiographic findings					
	Presence of vegetations	31 (86)	25 (81)	26 (72)	0.338
	Vegetation size in mm, median (IQR)	10 (7–15)	10 (6.5–15.5)	20 (10–25)	0.007
	Perivalvular abscess	4 (11)	15 (48)	0	<0.001
Complications					
	Heart failure	15 (42)	11 (36)	0	<0.001
	Renal failure	20 (56)	18 (58)	4 (11)	<0.001
	Systemic emboli	4 (11)	9 (29)	0	0.001
Antibiotic treatment					0.098
	Cloxacillin alone	1 (3)	1 (3)	6 (17)	
	Cloxacillin in combination^d^	13 (36)	8 (26)	16 (44)	
	Vancomycin alone	4 (11)	5 (16)	3 (8)	
	Vancomycin in combination^c^	15 (42)	16 (52)	11 (31)	
	Other antibiotics^e^	3 (8)	1 (3)	0	
Outcome					
	Surgical treatment	11 (31)	22 (71)	33 (92)	<0.001
	In-hospital mortality	16 (44)	14 (45)	2 (6)	<0.001
	1-year mortality	20 (56)	16 (52)	3 (8)	<0.001

Unless otherwise noted, all values are shown as n (%). Abbreviations: CoNS, coagulase-negative staphylococci; HIV, human immunodeficiency virus; ICD, intracardiac device; IE, infective endocarditis; IQR, interquartile range; NVE, native valve endocarditis; PCM/ICD, pacemaker/implantable cardioverter-defibrillator; PVE, prosthetic valve endocarditis; SD, standard deviation.

^a^Only 88 patients had available isolates; these 88 patients served as the basis for the rest of the study (see Fig 1).

^b^ *S*. *hominis* (7), *S*. *capitis* (2), *S*. *schleiferi* (2).

^c^PCM/ICD wire endocarditis was associated with tricuspid vegetations in 3 patients and aortic vegetations in 1 case.

^d^Administration of a second antibiotic, with or without a third, together with cloxacillin or vancomycin for >2 days.

^e^Two patients received teicoplanin, 1 received imipenem, and 1 received linezolid.

The clinical characteristics and outcomes of the 88 patients with available isolates are summarized by CoNS species in S1 Table. In-hospital and 1-year mortality rates were similar among groups but were highest among patients infected with *S*. *lugdunensis* (55% for both).

### CoNS Identification and Antibiotic Susceptibility Patterns

Ninety-eight isolates were identified in 88 patients: 70 *S*. *epidermidis* (71%), 11 *S*. *lugdunensis* (11%), 10 *S*. *hominis* (10%), and 7 other species (S2 Table). There were 6 and 4 patients with polymicrobial and polyclonal IE, respectively.

Susceptibility data are presented in S2 Table. Overall, 44% of the CoNS isolates were methicillin-resistant, including 51% of *S*. *epidermidis*. One-third of MRSE also were resistant to gentamicin, rifampin, and ciprofloxacin. All isolates of *S*. *lugdunensis* remained methicillin-susceptible.

Forty-two isolates had a vancomycin MIC ≥2 μg/mL (35 isolates at 2 μg/mL, 5 isolates at 3 μg/mL, and 2 isolates at 4 μg/mL). By species, 47% of the *S*. *epidermidis* isolates and 7% of other species (*P*<0.001) had vancomycin MICs of 2 μg/mL. The clinical, microbiological and therapeutic characteristics, as well as the outcomes, of the 39 patients with strains with vancomycin MIC ≥2 μg/mL can be seen in S3 Table. Two *S*. *epidermidis* isolates (both with vancomycin MICs of 3 μg/mL) demonstrated intermediate resistance to teicoplanin (MIC 16 μg/mL). We did not find any isolates with intermediate susceptibility to vancomycin. Vancomycin MIC did not show a trend towards increase over time for overall CoNS isolates (*P* = 0.49) nor for *S*. *epidermidis* specifically (*P* = 0.25); this pattern did not change according to methicillin susceptibility. All isolates remained linezolid- and daptomycin-susceptible, but 1 strain each of *S*. *epidermidis* and *S*. *capitis* had daptomycin MICs of 1.5 μg/mL. Regarding other recommended antibiotics for PVE, we found that 14/31 (45%) of the CoNS isolates in patients with PVE were resistant to gentamicin, rifampin and/or ciprofloxacin.

### Impact of Methicillin Susceptibility and Vancomycin MIC on Outcomes

Three of the 88 patients were excluded from the assessment of outcome because they were not treated with either cloxacillin or vancomycin, but instead were treated with teicoplanin (2) and linezolid (1). Cloxacillin was used in 38 (45%) patients (Group 1), and vancomycin was used in 47 (55%). Twelve of these 47 (26%) patients with MS-CoNS were treated with vancomycin because of penicillin allergy (3) or medical decision (9). Of these 12 patients, 2 died (17%). Among patients treated with vancomycin, 27 (32%) had CoNS isolates with vancomycin MICs <2 μg/mL (Group 2) and 20 (24%) had MICs ≥2 μg/mL (Group 3). The main clinical characteristics of the 3 groups are presented in Table 2.

**Table 2:** Clinical characteristics of 85 patients with CoNS IE according to treatment received (cloxacillin or vancomycin).^a^

			Treated with vancomycin		
		Treated with cloxacillin (N = 38)	Vancomycin MIC <2 (N = 27)	Vancomycin MIC ≥2 (N = 20)	*P* value
Mean age, y		68.5 (55–77)	66.0 (59–73.5)	60.5 (44.5–72.5)	0.182
Male gender		28 (74)	20 (74)	17 (85)	0.645
CoNS species					0.471
	*S*. *epidermidis*	22 (58)	19 (70)	16 (80)	
	*S*. *lugdunensis*	7 (18)	4 (15)	0	
	Other	6 (16)	3 (11)	2 (10)	
	Polymicrobial^b^	3 (8)	1 (4)	2 (10)	
Predisposing conditions and underlying diseases					
	Diabetes mellitus	3 (8)	3 (11)	4 (20)	0.387
	Chronic renal failure	2 (5)	2 (7)	2 (10)	0.865
	Hemodialysis	2 (5)	1 (4)	0	0.792
	History of cancer	4 (11)	1 (4)	4 (20)	0.184
	HIV infection	0	1 (4)	0	0.553
	Chronic liver disease	2 (5)	3 (11)	5 (25)	0.090
	Chronic lung disease	1 (3)	3 (11)	0	0.168
	Transplantation	0	1 (4)	1 (5)	0.500
	History of IE	1 (3)	0	1 (5)	0.713
Presumed mode of acquisition					0.080
	Nosocomial	5 (13)	7 (26)	6 (30)	
	Non-nosocomial healthcare associated	11 (29)	8 (30)	10 (50)	
	Community acquired	22 (58)	12 (44)	4 (20)	
Type of endocarditis, n (%)					0.109
	NV	11 (29)	10 (37)	7 (35)	
	PV	7 (18)	8 (30)	9 (45)	
	Pacemaker lead	20 (53)	9 (33)	4 (20)	
Valve involvement					0.301
	Aortic	6 (16)	9 (33)	7 (35)	
	Mitral	8 (21)	4 (15)	4 (20)	
	Tricuspid	0	0	1 (5)	
	PCM/ICD wire	20 (53)	9	4 (20)	
	Unknown	0	1 (4)	1 (5)	
	Mitral + aortic	4 (11)	2 (7)	2 (10)	
	Tricuspid + aortic	0	1 (4)	0	
	Tricuspid + aortic + mitral	0	1 (4)	1 (5)	
Echocardiogra-phic findings					
	Presence of vegetations	28 (74)	22 (82)	18 (90)	0.350
	Vegetation size in mm, median (IQR)	10 (0–19)	10 (1–16)	8 (2.5–10)	0.881
	Perivalvular abscess	5 (13)	5 (20)	5 (26)	0.436
Complications					
	Heart failure	9 (24)	9 (33)	5 (25)	0.670
	Renal failure	11 (29)	14 (52)	10 (50)	0.129
	Systemic emboli	3 (8)	1 (4)	4 (20)	0.178
Outcomes					
	Surgery	28 (74)	16 (59)	12 (60)	0.394
	In-hospital mortality	7 (18)	12 (44)	10 (50)	0.021
	1-year mortality	8 (21)	13 (48)	13 (65)	0.003

Unless otherwise noted, all values are shown as n (%). Abbreviations: CoNS, coagulase-negative staphylococci; HIV, human immunodeficiency virus; IE, infective endocarditis; IQR, interquartile range; PCM/ICD, pacemaker/implantable cardioverter-defibrillator; MIC, minimum inhibitory concentration; NV, native valve; PV, prosthetic valve.

^a^Three out of the 88 patients did not receive either cloxacillin or vancomycin and were not included.

^b^ *S*. *hominis* (7), *S*. *capitis* (2), *S*. *schleiferi* (2).

**Fig 2:**
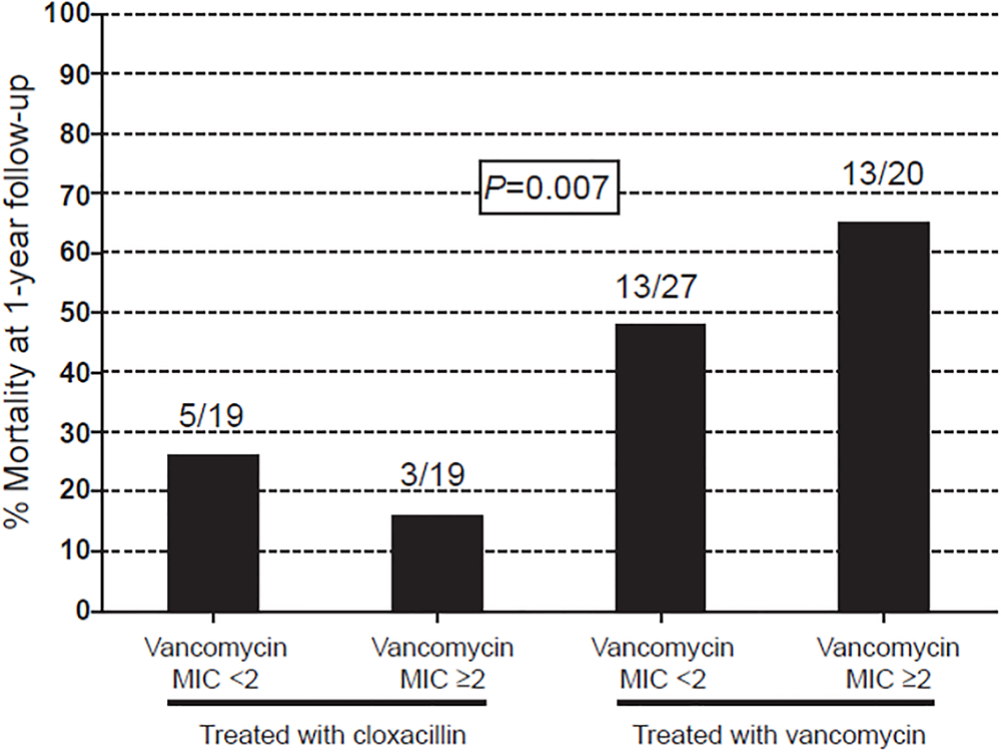
One-year mortality according to antibiotic treatment and vancomycin MIC. One-year mortality among 85 patients with coagulase-negative staphylococci infective endocarditis, according to antibiotic therapy and vancomycin minimum inhibitory concentration (MIC).

**Fig 3:**
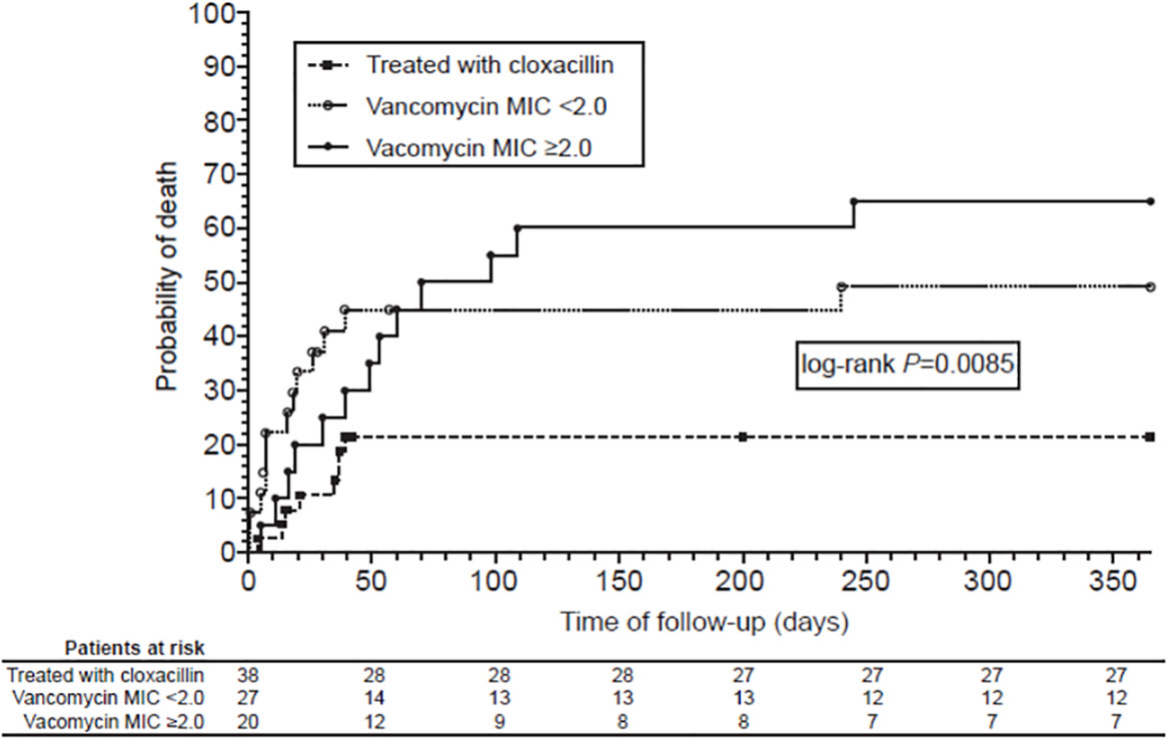
One-year survival analysis according to antibiotic therapy and vancomycin MIC. One-year survival analysis of 85 patients with coagulase-negative staphylococci infective endocarditis according to the treatment received and vancomycin minimum inhibitory concentration (MIC).

In-hospital mortality was higher among those patients treated with vancomycin, regardless of vancomycin MIC, than in those treated with cloxacillin (47% vs 18%; *P* = 0.012). In-hospital mortality was 18%, 44%, and 50% for Groups 1, 2, and 3, respectively (*P* = 0.021), and 1-year mortality was 21%, 48%, and 65%, respectively (*P* = 0.003). Mortality was similar for patients treated with cloxacillin regardless of vancomycin MIC (Fig 2). Conversely, the highest mortality was among vancomycin-treated patients infected with CoNS isolates having vancomycin MICs ≥2 μg/mL (*P* = 0.007). One-year survival analysis according to the treatment received and vancomycin MIC is presented in Fig 3.

Table 3 shows the univariate and multivariate analyses of risk factors associated with one-year mortality. Using the group of patients with MR-CoNS IE with vancomycin MICs <2 μg/mL treated with vancomycin (Group 2) as the reference, Group 1 (MS-CoNS IE treated with cloxacillin) and Group 3 (MR-CoNS IE with vancomycin MICs ≥2 μg/mL) respectively had independently lower and higher mortality (OR 0.12, 95% CI 0.02–0.55 and OR 3.7, 95% CI 0.9–15.2, respectively). Other independent variables associated with in-hospital mortality were heart failure and pacemaker lead endocarditis as a protective factor for mortality.

**Table 3:** Prognostic factors associated with one-year mortality in the 85 patients of CoNS endocarditis treated with either cloxacillin or vancomycin.

		Univariate analysis			Multivariate analysis		
		One-year mortality (N = 34)	Survived (N = 51)	*P* value	OR	95% CI	*P* value
Mean age (SD), y		63.5 (14.3)	62.6 (16.1)	0.795			
Male gender		25 (74)	40 (78)	0.602			
Year of diagnosis				0.929			
	1995	11 (32)	16 (31)				
	2000	12 (35)	20 (39)				
	2005	11 (32)	15 (29)				
CoNS species				0.773			
	*S*. *epidermidis*	22 (65)	35 (69)				
	*S*. *lugdunensis*	6 (18)	5 (10)				
	Other species^a^	4 (12)	7 (14)				
	Polymicrobial	2 (6)	4 (8)				
Predisposing conditions and underlying diseases							
	Diabetes mellitus	5 (15)	5 (10)	0.733			
	Chronic renal failure	4 (12)	2 (4)	0.212			
	Hemodialysis	1 (3)	2 (4)	1.000			
	History of cancer	3 (9)	6 (12)	0.735			
	HIV infection	1 (3)	0	0.400			
	Chronic liver disease	8 (24)	3 (6)	0.023			
	Chronic lung disease	1 (3)	3 (6)	0.647			
	Transplantation	0	1 (2)	1.000			
	History of IE	1 (3)	1 (2)	1.000			
Presumed mode of acquisition				0.619			
	Nosocomial	8 (24)	10 (20)				
	Non-nosocomial healthcare associated	13 (38)	16 (31)				
	Community acquired	13 (38)	25 (49)				
Type of endocarditis							
	NV	19 (56)	9 (18)	<0.001	0.1	0.02–0.51	0.006
	PV	13 (38)	11 (22)	0.094			
	Pacemaker lead	2 (6)	31 (61)	<0.001			
Valve involvement							
	Aortic	22 (65)	12 (24)	<0.001			
	Mitral	15 (44)	11 (22)	0.027			
	≥2 valves	8 (24)	3 (6)	0.023			
Complications							
	Heart failure	18 (53)	5 (10)	<0.001	6.2	1.5–25.2	0.011
	Renal failure	21 (62)	14 (28)	0.002			
	Systemic emboli	3 (9)	5 (10)	1.000			
	Perivalvular abscess	10 (29)	5 (10)	0.020			
Surgical treatment		16 (47)	40 (78)	0.003			
Treatment groups ^b^				0.003			
	Cloxacillin	8 (24)	30 (59)		0.12	0.02–0.55	0.008
	Vancomycin (MIC <2 μg/mL)	13 (38)	14 (27)		1.0		
	Vancomycin (MIC ≥2 μg/mL)	13 (38)	7 (14)		3.7	0.9–15.2	0.069

Unless otherwise noted, all values are shown as n (%). Abbreviations: CI, confidence interval; CoNS, coagulase-negative staphylococci; HIV, human immunodeficiency virus; IE, infective endocarditis; MIC, minimum inhibitory concentration; NV, native valve; OR, odds ratio; PV, prosthetic valve; SD, standard deviation.

^a^ *S*. *hominis* (7), *S*. *capitis* (2), *S*. *schleiferi* (2).

^b^In the multivariable regression analysis, cloxacillin treatment is the reference category. Vancomycin MIC <2 μg/mL effect on one-year mortality is compared to cloxacillin and Vancomycin MIC ≥2 μg/mL is compared to Vancomycin MIC <2 μg/mL.

## Discussion

### Incidence, Types of IE, and Clinical Characteristics

CoNS are increasingly identified as a cause of NVE [8, 21] and caused 9% of all NVE in our cohort. In one recent multicenter investigation, nearly 8% of all NVE not associated with intravenous drug use (IVDU) were caused by CoNS, predominantly *S*. *epidermidis* [8]. A recent multinational prospective cohort study found that 16% of PVE not due to IVDU was attributed to CoNS [6]. *S*. *epidermidis* was isolated in 82% of cases, and 67% of these were methicillin-resistant. These results are similar to those seen in our series. In recent data provided by the International Collaboration on Endocarditis (ICE), CoNS was shown to be the second global cause of ICD IE immediately after *S*. *aureus*, being more often nosocomially-acquired than *S*.*aureus* [18].

Overall, we noted 44% of CoNS isolates were methicillin-resistant, a lower rate than documented in other surveys [19–22], and we did not find an association between methicillin-resistance and healthcare acquisition as did previous studies [8, 21]. Reduced susceptibility to vancomycin (MIC >2 μg/mL) was found in 3% of CoNS isolates in our study, similar to a 2% rate in a recent review of bloodstream infections caused by CoNS [23]. As was the case with other recent summaries of susceptibility data from Spain [20–22], no CoNS isolates were resistant to vancomycin, linezolid, or daptomycin.

### Impact of Oxacillin Susceptibility and Vancomycin MIC on Outcome

Vancomycin treatment was associated with higher mortality, especially among patients with IE due to isolates with vancomycin MICs ≥2 μg/mL. A number of studies have found a correlation between vancomycin MIC and poorer outcomes among patients with methicillin-resistant *S*. *aureus* bacteremia [24]. The present study is, to our knowledge, the first to demonstrate the same correlation between poor outcomes and vancomycin MIC in CoNS IE. This finding could have important clinical implications In addition, it suggests an important role for alternative antibacterial agents. In our series, higher vancomycin MICs had no impact on outcomes in patients with MS-CoNS IE receiving cloxacillin. In contrast, a recent study showed that among patients with methicillin-susceptible *S*. *aureus* (MSSA) bacteremia treated with flucloxacillin, outcomes were less favourable among those with higher vancomycin MICs [25]. Our group recently found the same association of high vancomycin MIC and left-sidedMSSA IE [26]. Higher vancomycin MICs could be a marker of bad prognosis in *S*. *aureus* bacteremia and IE regardless of the administered treatment, with the causative mechanism yet to be identified, but in light of our results, however, we cannot conclude that this hypothetical mechanism is common to all staphylococci.

### Impact of Antibiotic Treatment on Outcome

Vancomycin monotherapy is the treatment of choice for MR-CoNS NVE [10,27]. However, we found very high mortality rates in vancomycin-treated patients. A vancomycin trough of 15–20 μg/mL is supposed to achieve the suggested target AUC/MIC ratios of ≥400 for organisms with MICs ≤1 μg/mL [28]. For patients infected with CoNS isolates having MICs ≥2 μg/mL, this ratio was likely not achieved in most cases; this may explain why patients with MR-CoNS IE with vancomycin MIC ≥2 μg/mL had the highest mortality rates. At the time the AHA guidelines were published, higher vancomycin troughs were not yet recommended and so were not targeted in our patients. Nonetheless, Holmes et al [29] did not clearly find better outcomes in patients with *S*. *aureus* bacteremia achieving an AUC/MIC ratio >400. Thus, given these recent data and a lack of evidence regarding a correlation between AUC/MIC and CoNS bacteremia/IE outcomes, our results suggest the use of alternative agents to vancomycin.

For cases of MR-CoNS PVE, the recommended therapy is vancomycin in combination with rifampin and gentamicin [10,27]. Interestingly, we found that 45% of the CoNS causing PVE were resistant to at least one recommended drug associated to vancomycin (data not shown). Thus, in our setting, it may not be unusual that empirical treatment for CoNS-PVE is inappropriate. Therefore, better antibiotic options are needed for CoNS PVE since deciding the most suitable combination for CoNS PVE may result challenging in light of current evidence. Although clinical data supporting the use of ceftaroline for CoNS IE is lacking, some *in vitro* studies provide interesting results, showing a good susceptibility profile for ceftaroline against CoNS that includes methicillin-resistant, linezolid-resistant and daptomycin non-susceptible strains [30,31]. Besides, no emergence of ceftaroline-resistant strains has been described to date. Clinical evidence also lacks for telavancin, whose *in vitro* activity against CoNS is better than vancomycin due to its dual mechanism of action, which confers a rapid bactericidal activity [32, 33]. However, increased MICs for telavancin have been reported in some strains of *S*. *epidermidis* with reduced susceptibility to glycopeptides [33]. As occurred with the former agent, no clinical experience with tigecycline for the treatment of CoNS IE is yet available and experimental evidence from the IE model has been neither published. Linezolid use is limited in monotherapy due to its bacteriostatic activity. Noteworthy, emergence of resistance to linezolid among CoNS is increasingly reported [34]. Results derived from both *in vivo* studies and clinical experience with daptomycin are encouraging [35–38]. We have previously shown in animal models that daptomycin was a better therapeutic option than vancomycin [35, 39], particularly for IE caused by MR-CoNS with vancomycin MICs >1 μg/mL. Consequently, we believe that daptomycin should be considered as the preferred alternative for patients with NVE or PVE caused by MR-CoNS.

### Study Limitations

This study has several limitations. First, it is a non-randomized, observational study. Second, it was conducted at a single, tertiary referral center for IE, so referral bias limits external validity, as does the loss of some of the isolates of transferred patients. Third, while the total number of IE cases diagnosed in our center is large, the number of documented CoNS cases is relatively small and did not allow us to investigate the impact of vancomycin MIC in subpopulations of interest (eg, at the species level, according to IE type). As stated above, our study lacks pharmacokinetic and pharmacodynamic data, especially regarding the assessment of AUC/MIC ratios.

## Conclusion

CoNS are well recognized as an important cause of IE, including infections of both native and prosthetic valves, as well as those involving pacemakers. Such infections are often acquired in healthcare settings, and are caused increasingly by pathogens less susceptible to agents like vancomycin that have long been standards of care. While several studies have documented poor outcomes among vancomycin-treated patients with serious *S*. *aureus* infections caused by isolates with higher vancomycin MICs, our report is the first to demonstrate a similar pattern among patients with MR-CoNS IE. Alternatives to currently recommended drugs should be considered in future studies. So, additional randomized, controlled studies are warranted.

## Supporting Information

S1 Table Clinical characteristics and outcomes of 88 patients with IE due to CoNS, according to the CoNS species. (DOC)

S2 Table Activity of 11 selected antibiotics as determined by Etest for 98 CoNS isolates from 88 patients with IE. (DOC)

S3 Table Basal characteristics, microbiological features and outcome of the 39 patients with CoNS strains with vancomycin ≥2 μg/mL. (DOC)
