# Supplementary material for: Epidemiology and Prognosis of Coagulase-Negative Staphylococcal Endocarditis: Impact of Vancomycin Minimum Inhibitory Concentration
Source: PLoS One. 2015 May 11;10(5):e0125818. doi: 10.1371/journal.pone.0125818 (PMC4427314; doi:10.1371/journal.pone.0125818)
Supplement: S1 Table — (DOC) [file pone.0125818.s001.doc]

**Table S1**. Clinical characteristics and outcomes of 88 patients with IE due to CoNS, according to the CoNS species.

|  |  | *S. epidermidis*^a^  (N=60) | *S. lugdunensis*  (N=11) | Other CoNS species^b^  (N=11) | Polymicrobial^c^  (N=6) |
| --- | --- | --- | --- | --- | --- |
| Mean age (SD), y |  | 60.9 (16.4) | 70.0 (12.8) | 63.0 (11.3) | 63.0 (17.8) |
| Male gender |  | 47 (78) | 7 (64) | 8 (73) | 6 (100) |
| Year of diagnosis |  |  |  |  |  |
|  | 1995-1999 | 14 (23) | 6 (55) | 4 (36) | 3 (50) |
|  | 2000-2004 | 26 (43) | 3 (27) | 2 (18) | 2 (33) |
|  | 2005-2008 | 20 (33) | 2 (18) | 5 (46) | 1 (17) |
| β-lactam susceptibility | Penicillin-susceptible | 2 (3) | 8 (73) | 2 (18) | 0 |
|  | Methicillin-susceptible | 29 (48) | 11 (100) | 7 (64) | 2 (33) |
| Antibiotic resistance (oxacillin-susceptible) |  |  |  |  |  |
|  | G + R + C susceptible | 25 (86) | 11 (100) | 6 (86) | 2 (100) |
|  | G resistant | 2 (7) | 0 | 1 (14) | 0 |
|  | R resistant | 1 (3) | 0 | 0 | 0 |
|  | G + C resistant | 1 (3) | 0 | 0 | 0 |
| Antibiotic resistance among MR isolates |  |  |  |  |  |
|  | G + C resistant | 10 (32) | 0 | 1 (25) | 2 (50) |
|  | G + R + C susceptible | 4 (13) | 0 | 2 (50) | 0 |
|  | G resistant | 3 (10) | 0 | 0 | 0 |
|  | C resistant | 1 (3) | 0 | 0 | 0 |
|  | G + R resistant | 9 (29) | 0 | 1 (25) | 2 (50) |
|  | G + C resistant | 1 (3) | 0 | 0 | 0 |
|  | G + R + C resistant | 3 (10) | 0 | 0 | 0 |
| Predisposing conditions and underlying diseases |  |  |  |  |  |
|  | Diabetes mellitus | 9 (15) | 1 (9) | 0 | 1 (17) |
|  | Chronic renal failure | 6 (10) | 0 | 0 | 0 |
|  | Hemodialysis | 3 (5) | 0 | 0 | 0 |
|  | History of cancer | 5 (8) | 1 (9) | 1 (9) | 2 (33) |
|  | HIV infection | 1 (2) | 0 | 0 | 0 |
|  | Chronic liver disease | 5 (8) | 0 | 5 (46) | 1 (17) |
|  | Chronic lung disease | 2 (3) | 2 (18) | 0 | 0 |
|  | Transplantation | 2 (3) | 0 | 0 | 0 |
|  | History of IE | 0 | 0 | 2 (18) | 0 |
| Presumed mode of acquisition |  |  |  |  |  |
|  | Nosocomial | 14 (23) | 1 (9) | 2 (18) | 1 (17) |
|  | Non-nosocomial healthcare associated | 23 (38) | 2 (18) | 4 (36) | 1 (17) |
|  | Community acquired | 23 (38) | 8 (73) | 5 (46) | 4 (66) |
| Type of endocarditis |  |  |  |  |  |
|  | NV | 17 (28) | 4 (36) | 4 (36) | 4 (67) |
|  | PV | 18 (30) | 2 (18) | 4 (36) | 1 (17) |
|  | Pacemaker lead | 25 (42) | 5 (46%) | 3 (27) | 1 (17) |
| Valve involvement |  |  |  |  |  |
|  | Aortic | 14 (23) | 4 (36) | 3 (27) | 2 (33) |
|  | Mitral | 11 (18) | 2 (18) | 2 (18) | 1 (17) |
|  | Tricuspid | 1 (2) | 0 | 0 | 0 |
|  | PCM/ICD wire | 25 (42) | 5 (46) | 3 (27) | 1 (17) |
|  | Unknown | 1 (2) | 0 | 1 (10) | 0 |
|  | Mitral + aortic | 4 (7) | 0 | 2 (18) | 2 (33) |
|  | Tricuspid + aortic | 2 (3) | 0 | 0 | 0 |
|  | Tricuspid + aortic + mitral | 2 (3) | 0 | 0 | 0 |
| Echocardiographic findings |  |  |  |  |  |
|  | Presence of vegetations | 47 (78) | 10 (91) | 7 (64) | 5 (83) |
|  | Vegetation size in mm, median (IQR) | 13 (9-20) | 10 (10-23) | 14 (10-25) | 13.5 (5-20) |
|  | Perivalvular abscess | 10 (18) | 2 (20) | 2 (18) | 1 (17) |
| Complications |  |  |  |  |  |
|  | Heart failure | 13 (22) | 3 (27) | 6 (55%) | 2 (33) |
|  | Renal failure | 23 (38) | 4 (36) | 6 (55) | 4 (67) |
|  | Systemic emboli | 6 (10) | 0 | 1 (9) | 2 (33) |
| Antibiotic treatment |  |  |  |  |  |
|  | Cloxacillin alone | 5 (8) | 0 | 1 (9) | 0 |
|  | Cloxacillin in combination^d^ | 17 (28) | 7 (64) | 5 (46) | 3 (50) |
|  | Vancomycin alone | 7 (12) | 1 (9) | 1 (9) | 0 |
|  | Vancomycin in combination^d^ | 29 (48) | 3 (27) | 4 (36) | 2 (33) |
|  | Other antibiotics^e^ | 2 (3) | 0 | 0 | 1 (17) |
| Outcome |  |  |  |  |  |
|  | Surgical treatment | 39 (65) | 7 (64) | 7 (64) | 4 (67) |
|  | In-hospital mortality | 21 (35) | 6 (55) | 4 (36) | 0 |
|  | 1-year mortality | 24 (40) | 6 (55) | 4 (36) | 2 (33) |

Unless otherwise noted, all values are shown as n (%). Abbreviations: C, ciprofloxacin; CoNS, coagulase-negative staphylococci; G, gentamicin; HIV, human immunodeficiency virus; IE, infective endocarditis; IQR, interquartile range; MIC, minimum inhibitory concentration; MR, methicillin-resistant; NV, native valve; PCM/ICD, pacemaker/implantable cardioverter-defibrillator; PV, prosthetic valve; R, rifampin; SD, standard deviation.

^a^Four patients of *S. epidermidis* endocarditis were caused by polyclonal isolates. In these patients, the strain with the higher vancomycin MIC was selected for analysis.

^b^*S. hominis* (7), *S. capitis* (2), *S. schleiferi* (2).

^c^*S. epidermidis* + *S. hominis* (3), *S. epidermidis* + *S. haemolyticus* (2) and *S. epidermidis* + *S. warneri* (1).

^d^Administration of a second antibiotic, with or without a third, together with cloxacillin or vancomycin for >2 days. ^e^Two patients received teicoplanin and one received linezolid.
