# Supplementary material for: Epidemiology and Prognosis of Coagulase-Negative Staphylococcal Endocarditis: Impact of Vancomycin Minimum Inhibitory Concentration
Source: PLoS One. 2015 May 11;10(5):e0125818. doi: 10.1371/journal.pone.0125818 (PMC4427314; doi:10.1371/journal.pone.0125818)
Supplement: S2 Table — (DOC) [file pone.0125818.s002.doc]

**Table S2**. Activity of 11 selected antibiotics as determined by Etest for 98 CoNS isolates from 88 patients with IE.

| **Antimicrobial agent** | **MIC_50_** | **MIC_90_** | **Range** | **CLSI^a^**  **%S / %I / %R** |
| --- | --- | --- | --- | --- |
| ***S. epidermidis* (n=70)** |  |  |  |  |
| Oxacillin | 0.5 | 256 | 0.06-256 | 49 / 0 / 51 |
| Clindamycin | 0.12 | 256 | 0.06-256 | 73 / 0 / 27 |
| Erythromycin | 0.38 | 256 | 0.12-256 | 54 / 0 / 46 |
| TMP/SMX | 0.09 | 32 | 0.016-256 | 83 / 0 / 17 |
| Gentamicin | 0.12 | 48 | 0.023-256 | 69 / 0 / 32 |
| Rifampin | 0.008 | 0.016 | 0.003-32 | 91 / 0 / 9 |
| Ciprofloxacin | 0.19 | 32 | 0.06-32 | 73 / 0 / 27 |
| Vancomycin | 2 | 2 | 0.5-4 | 100 / 0 / 0 |
| Teicoplanin | 2 | 6 | 0.12-16 | 97 / 3 / 0 |
| Daptomycin | 0.5 | 0.75 | 0.06-1.5 | 100 / 0 / 0 |
| Linezolid | 1 | 2 | 0.38-2 | 100 / 0 / 0 |
| ***S. lugdunensis* (n=11)** |  |  |  |  |
| Oxacillin | 0.5 | 1 | 0.25-3 | 100 / 0 / 0 |
| Clindamycin | 0.09 | 0.12 | 0.06-0.19 | 100 / 0 / 0 |
| Erythromycin | 0.09 | 0.12 | 0.03-0.19 | 100 / 0 / 0 |
| TMP/SMX | 0.09 | 0.12 | 0.05-0.12 | 100 / 0 / 0 |
| Gentamicin | 0.06 | 0.09 | 0.03-0.09 | 100 / 0 / 0 |
| Rifampin | 0.004 | 0.008 | 0.003-0.008 | 100 / 0 / 0 |
| Ciprofloxacin | 0.25 | 1 | 0.12-1 | 100 / 0 / 0 |
| Vancomycin | 1 | 1.5 | 0.75-1.5 | 100 / 0 / 0 |
| Teicoplanin | 0.25 | 0.5 | 0.25-1 | 100 / 0 / 0 |
| Daptomycin | 0.25 | 0.38 | 0.19-0.38 | 100 / 0 / 0 |
| Linezolid | 0.75 | 1.5 | 0.5-1.5 | 100 / 0 / 0 |
| ***S. hominis* (n=10)** |  |  |  |  |
| Oxacillin | 0.12 | 3 | 0.09-4 | 60 / 0 / 40 |
| Clindamycin | 0.06 | 256 | 0.016-256 | 70 / 0 / 30 |
| Erythromycin | 0.25 | 256 | 0.016-256 | 60 / 0 / 40 |
| TMP/SMX | 0.09 | 6 | 0.023-6 | 80 / 0 / 20 |
| Gentamicin | 0.06 | 32 | 0.023-32 | 60 / 0 / 40 |
| Rifampin | 0.06 | 0.12 | 0.004-2 | 100 / 0 / 0 |
| Ciprofloxacin | 0.25 | 32 | 0.09-32 | 80 / 0 / 20 |
| Vancomycin | 1 | 2 | 0.75-2 | 100 / 0 / 0 |
| Teicoplanin | 0.25 | 3 | 0.12-6 | 100 / 0 / 0 |
| Daptomycin | 0.25 | 0.75 | 0.12-0.75 | 100 / 0 / 0 |
| Linezolid | 1 | 1.5 | 1-2 | 100 / 0 / 0 |
| **Other CoNS^b^ (n=7)** |  |  |  |  |
| Oxacillin | 0.25 | 256 | 0.09-256 | 4 / 0 / 3 |
| Clindamycin | 0.19 | 256 | 0.06-256 | 5 / 0 / 2 |
| Erythromycin | 0.19 | 256 | 0.06-256 | 5 / 0 / 2 |
| TMP/SMX | 0.06 | 32 | 0.03-32 | 6 / 0 / 1 |
| Gentamicin | 0.06 | 32 | 0.03-32 | 5 / 0 / 2 |
| Rifampin | 0.008 | 0.016 | 0.006-0.016 | 7 / 0 / 0 |
| Ciprofloxacin | 0.19 | 32 | 0.12-32 | 6 / 0 / 1 |
| Vancomycin | 1.5 | 4 | 1-4 | 7 /0 /0 |
| Teicoplanin | 1 | 6 | 0.75-6 | 7 /0 /0 |
| Daptomycin | 0.5 | 1.5 | 0.09-1.5 | 7 / 0 / 0 |
| Linezolid | 0.75 | 2 | 0.5-2 | 7 / 0 / 0 |

Abbreviations: CLSI, Clinical and Laboratory Standards Institute; CoNS, coagulase-negative staphylococci; I, intermediate; IE, infective endocarditis; R, resistant; S, susceptible; TMP/SMX, trimethoprim/sulfamethoxazole.

^a^Criteria as published by the Clinical and Laboratory Standards Institute, 2010.

^b^For groups with <10 samples, % S/I/R are not calculated. Susceptibility shown as number of isolates in each category. CoNS species were *S. schleiferi* (2), *S. capitis* (2), *S. haemolyticus* (2), and *S. warneri* (1).
