# Supplementary material for: Epidemiology and Prognosis of Coagulase-Negative Staphylococcal Endocarditis: Impact of Vancomycin Minimum Inhibitory Concentration
Source: PLoS One. 2015 May 11;10(5):e0125818. doi: 10.1371/journal.pone.0125818 (PMC4427314; doi:10.1371/journal.pone.0125818)
Supplement: S3 Table — (DOC) [file pone.0125818.s003.doc]

**Table S3.** Basal characteristics, microbiological features and outcome of the 39 patients with CoNS strains with vancomycin ≥2 µg/mL

| Case # | Age/  Gender | Acquisition | Type of IE | Species | Vancomycin MIC (µg/mL) | Susceptibility to Oxacillin | # Antibiotic  Resistances* | Surgery | Antibiotic treatment | Outcome at one-year |
| --- | --- | --- | --- | --- | --- | --- | --- | --- | --- | --- |
| 1 | 54/F | Community | Native | SEPI | 2.0 | S | 5 | Y | Cloxacillin | Alive |
| 2 | 58/M | Community | ICD | SEPI | 2.0 | S | 1 | Y | Cloxacillin | Alive |
| 3 | 53/M | Community | ICD | SEPI | 3.0 | S | 0 | Y | Cloxacillin | Alive |
| 4 | 74/M | HCR-NN | Prosthetic | SEPI | 3.0 | S | 0 | N | Cloxacillin | Alive |
| 5 | 69/F | HCR-NN | Native | SEPI | 2.0 | R | 6 | N | Vancomycin | Death |
| 6 | 50/M | HCR-NN | Prosthetic | SEPI | 2.0 | R | 1 | Y | Vancomycin | Alive |
| 7 | 31/F | HCR-NN | ICD | SEPI | 2.0 | S | 0 | Y | Cloxacillin | Alive |
| 8 | 37/M | HCR-NN | Native | SEPI | 2.0 | R | 6 | N | Vancomycin | Death |
| 9 | 54/M | HCR-NN | Prosthetic | SEPI | 2.0 | R | 5 | Y | Vancomycin | Death |
| 10 | 72/M | Community | ICD | SEPI | 2.0 | S | 0 | Y | Cloxacillin | Alive |
| 11 | 79/M | Community | Native | SEPI | 2.0 | S | 1 | Y | Cloxacillin | Alive |
| 12 | 79/M | Community | Native | SEPI | 2.0 | S | 0 | N | Cloxacillin | Death |
| 13 | 49/M | HCR-NN | ICD | SEPI | 2.0 | S | 0 | Y | Cloxacillin | Alive |
| 14 | 79/M | Community | ICD | SEPI | 2.0 | S | 0 | Y | Cloxacillin | Alive |
| 15 | 64/M | HCR-N | Native | SEPI | 3.0 | R | 6 | N | Vancomycin | Death |
| 16 | 73/M | Community | ICD | SSCH | 2.0 | S | 0 | Y | Vancomycin | Alive |
| 17 | 77/M | HCR-N | Native | SHAE | 4.0 | R | 6 | N | Vancomycin | Alive |
| 18 | 67/M | HCR-N | Native | SEPI | 2.0 | S | 0 | N | Cloxacillin | Alive |
| 19 | 53/F | HCR-NN | Prosthetic | SEPI | 2.0 | R | 2 | N | Vancomycin | Death |
| 20 | 37/M | HCR-NN | Native | SEPI | 2.0 | R | 5 | Y | Vancomycin | Death |
| 21 | 77/M | Community | Prosthetic | SEPI | 2.0 | R | 3 | Y | Vancomycin | Death |
| 22 | 76/M | HCR-NN | Native | SEPI | 2.0 | S | 2 | N | Cloxacillin | Alive |
| 23 | 57/M | HCR-NN | Prosthetic | SEPI | 2.0 | S | 5 | Y | Vancomycin | Alive |
| 24 | 73/F | HCR-NN | Prosthetic | SEPI | 2.0 | R | 3 | Y | Vancomycin | Death |
| 25 | 74/M | HCR-N | ICD | SEPI | 2.0 | R | 2 | Y | Vancomycin | Alive |
| 26 | 72/M | HCR-NN | Native | SEPI | 2.0 | R | 3 | N | Vancomycin | Death |
| 27 | 75/M | Community | Native | SEPI | 2.0 | S | 0 | Y | Cloxacillin | Death |
| 28 | 55/M | HCR-NN | Prosthetic | SEPI | 2.0 | R | 2 | Y | Vancomycin | Death |
| 29 | 38/M | Community | Prosthetic | SHOM | 2.0 | R | 6 | Y | Vancomycin | Death |
| 30 | 70/M | HCR-NN | Native | SHOM | 2.0 | S | 0 | Y | Cloxacillin | Alive |
| 31 | 64/M | HCR-N | Native | SEPI | 2.0 | R | 3 | N | Vancomycin | Death |
| 32 | 69/F | HCR-N | Native | SEPI | 2.0 | S | 1 | N | Cloxacillin | Alive |
| 33 | 39/M | Community | ICD | SEPI | 2.0 | R | 3 | Y | Vancomycin | Alive |
| 34 | 84/M | HCR-N | ICD | SEPI | 2.0 | S | 0 | Y | Cloxacillin | Alive |
| 35 | 16/M | HCR-N | ICD | SEPI | 3.0 | R | 2 | Y | Vancomycin | Alive |
| 36 | 77/F | HCR-NN | ICD | SEPI | 3.0 | S | 2 | Y | Cloxacillin | Alive |
| 37 | 62/M | Community | ICD | SEPI | 2.0 | S | 1 | Y | Cloxacillin | Alive |
| 38 | 86/M | Community | ICD | SEPI | 2.0 | R | 1 | Y | Cloxacillin | Death |
| 39 | 66/M | HCR-N | Prosthetic | SEPI | 2.0 | R | 4 | N | Vancomycin | Death |

IE= Infective endocarditis; F= female; M=male; ICD=intracardiac device; HCR-N=healthcare-related, nosocomial; HCR-NN=healthcare-related, non-nosocomial; MIC= Minimum inhibitory concentration; SEPI= *S. epidermidis*; SHOM= *S. hominis*; SSCH= *S. schleiferi*; SHAE= *S. haemolyticus*; R= resistant; S= susceptible; N= no; Y=yes.

* Number of antibiotic resistances including resistance to vancomycin, oxacillin, clindamycin, erythromycin, trimethroprim-sulfametoxazol, gentamicin, rifampin and ciprofloxacin.
